# Small-cell lung cancer transformation from EGFR-mutant adenocarcinoma after EGFR-TKIs resistance: A case report

**DOI:** 10.1097/MD.0000000000026911

**Published:** 2021-08-13

**Authors:** Yiqian Jiang, Leyi Shou, Qingmin Guo, Yanhong Bao, Xiaoping Xu, Suhong An, Jianfeng Lu

**Affiliations:** a Department of Radiotherapy, Xiaoshan Affiliated Hospital of Wenzhou Medical University, Hangzhou, Zhejiang, China; b Department of Pathology, Xiaoshan Affiliated Hospital of Wenzhou Medical University, Hangzhou, Zhejiang, China; c Department of Infectious Disease, Xiaoshan Affiliated Hospital of Wenzhou Medical University, Hangzhou, Zhejiang, China.

**Keywords:** epidermal growth factor receptor-mutant adenocarcinoma, small-cell lung cancer, transformation

## Abstract

**Rationale:**

With the recent advancements in molecular biology research, epidermal growth factor receptor-tyrosine kinase inhibitors (EGFR-TKIs) have emerged as excellent therapies for patients with EGFR-mutant cancers. However, these patients inevitably develop cross-acquired resistance to EGFR-TKIs. Transformation to small-cell lung cancer (SCLC) is considered a rare resistance mechanism against EGFR-TKI therapy. Here, we report a case of TKI resistance due to SCLC transformation and demonstrate its mechanisms and clinical features.

**Patient concerns:**

A 54-year-old Chinese man with a history of smoking for 40 years complained of an intermittent cough in March 2019.

**Diagnosis:**

Transbronchial lung biopsy was performed on the basal segment of the left lower lobe, which confirmed lung adenocarcinoma. In January 2020, repeat biopsy was performed, and the results of immunohistochemistry (IHC) staining showed TTF-1 (+), CK7 (+), napsin A (+), syn (+), and CD56 (+), with a Ki-67 (+) index 80% of small cell carcinomas. Infiltrating adenocarcinomas and small cell carcinomas were observed.

**Interventions:**

Icotinib (125 mg thrice daily) was administered as a first-line treatment from June 2019. We subsequently administered a chemotherapy regimen consisting of etoposide (180 mg, days 1–3) plus cisplatin (45 mg, days 1–3) every 3 weeks for 1 cycle after recurrence. As the patient could not tolerate further chemotherapy, he continued taking icotinib orally and received whole-brain radiotherapy 10 times to a total dose of 30 Gy after brain metastases.

**Outcomes:**

The patient relapsed after successful treatment with icotinib for 9 months. A partial response was achieved after 4 cycles of chemotherapy, and despite the brief success of chemotherapy, our patient exhibited brain metastasis and metastases of the eleventh thoracic spine and the second lumbar vertebra with pathological fracture. The patient eventually died of aggressive cancer progression.

**Lessons:**

Our case highlights the possibility of SCLC transformation from EGFR-mutant adenocarcinoma and the importance of repeat biopsy for drug resistance. Serum neuron-specific enolase levels may also be useful for detecting early SCLC transformation.

## 1 Introduction

Epidermal growth factor receptor (EGFR) -tyrosine kinase inhibitors (TKIs) have revolutionized the treatment of patients with EGFR-mutant non-small cell lung cancer (NSCLC). However, these patients inevitably develop resistance to EGFR-TKIs after a median time of approximately 6 to 8 months.^[1,2]^ Numerous resistance mechanisms have been discovered to date;^[3]^ notably, the EGFR T790M mutation,^[4]^ MET amplification,^[5]^ and histologic transformation including small cell lung cancer (SCLC) transformation^[6]^ have all been suggested as common possible mechanisms of TKI resistance. Similarly, transformation to SCLC has been defined as a rare mechanism occurring in approximately 3.5% of patients.^[7]^ Here, we report a case of TKI resistance due to SCLC transformation, and demonstrate its mechanisms and clinical features.

## 2 Case presentation

**Figure 1:**
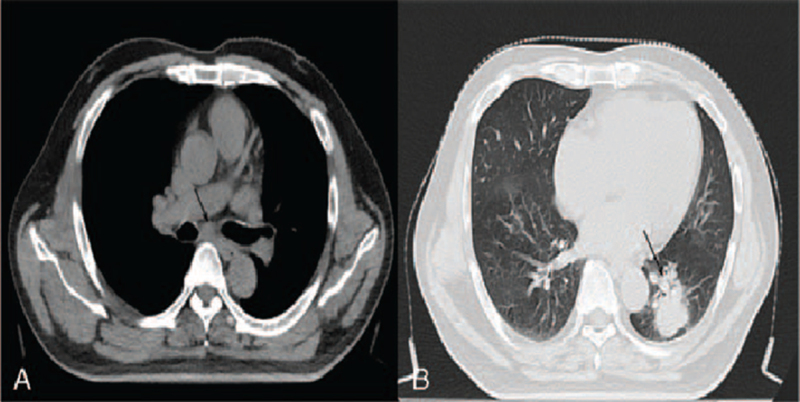
Baseline of the left lower lobe mass (1A) and a clear mediastinal structure with enlarged lymph nodes (1B) (March 2019).

**Figure 2:**
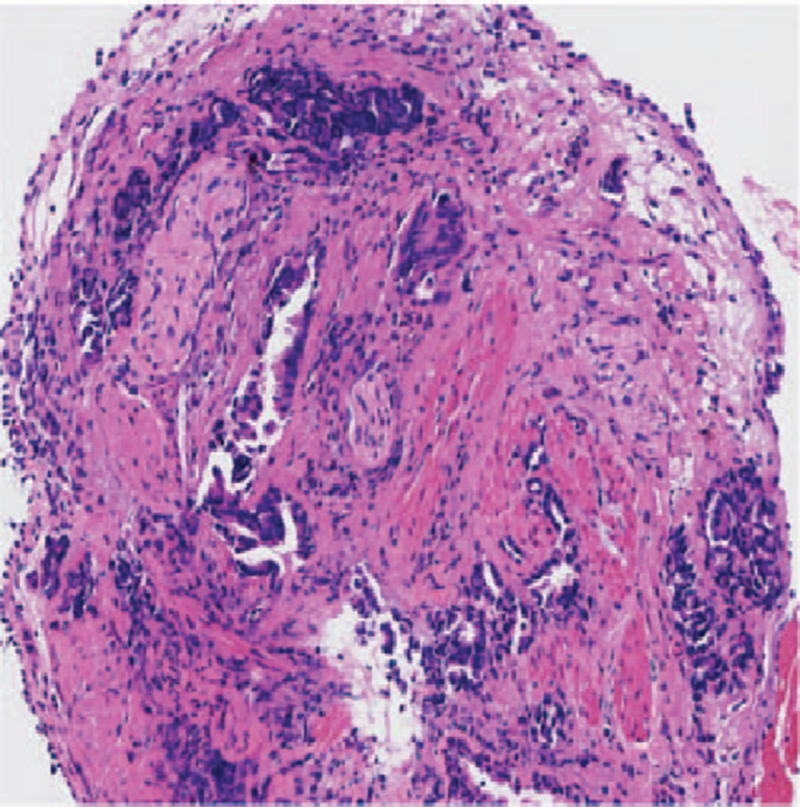
The pathological diagnosis of biopsy was lung adenocarcinoma ×100 H&E (hematoxylin and eosin).

A 54-year-old Chinese man with a history of smoking presented to our department in March 2019 complaining of intermittent cough. Computed tomography (CT) revealed a 4.0 × 3.5 cm mass in the left lower lobe and enlarged mediastinal lymph nodes (Fig. 1). Positron emission tomography 18/CT scan revealed a mass in the left lower lobe with intense uptake of (18F) fluorodeoxyglucose and hypermetabolic mediastinal lymph nodes. Meanwhile, a hypermetabolic right adrenal gland was also identified. Transbronchial lung biopsy was subsequently performed on the basal segment of the left lower lobe, which confirmed lung adenocarcinoma (Fig. 2). Next-generation sequencing was employed to detect EGFR exon 19 deletion. Subsequently, the patient was diagnosed with stage IV lung adenocarcinoma with an EGFR exon 19 deletion mutation (cT2N2M1 according to TNM classification, version 8).

**Figure 3:**
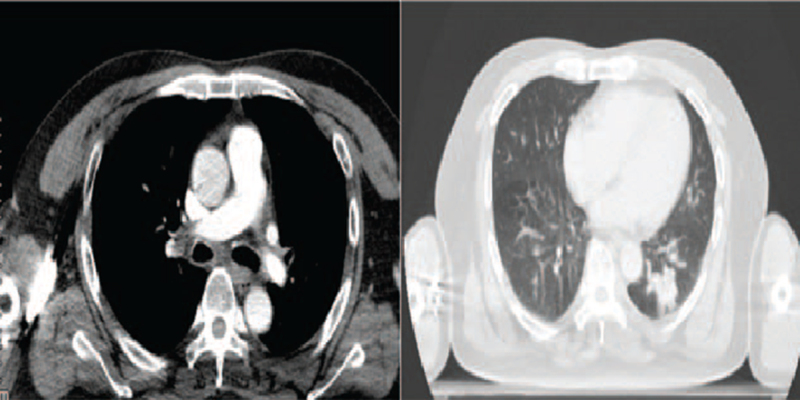
Partial remission upon gefitinib treatment (July 2019).

**Figure 4:**
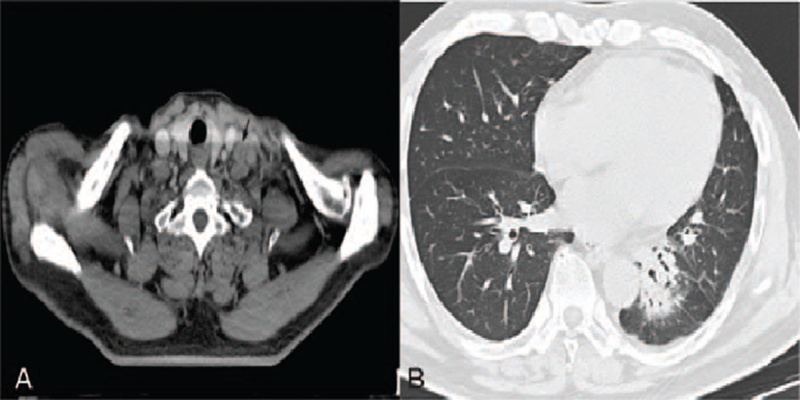
Enlarged supraclavicular lymph node (4A) and an enlarged left lower lobe mass (4B) (December 2019).

**Figure 5:**
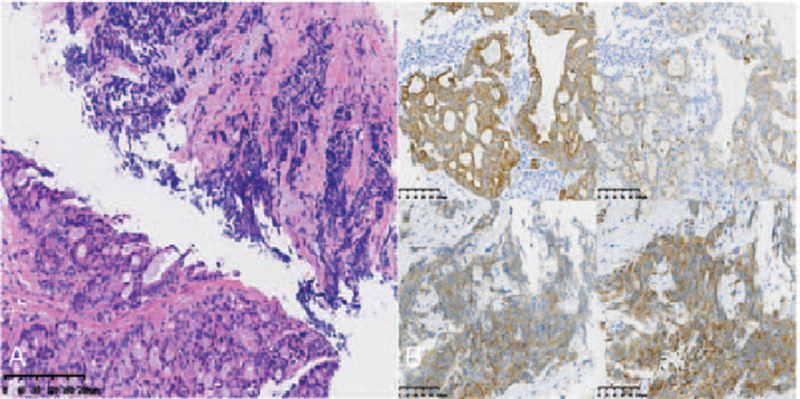
The pathological diagnosis of re-biopsy was mixed adenocarcinoma and neuroendocrine carcinoma (5A) ×100 H&E. Immunohistochemistry staining: CK7, Napsin A, CD56, Syn was positive (5B) ×200 CK7, Napsin A, CD56, Syn IHC (immunological histological chemistry).

**Figure 6:**
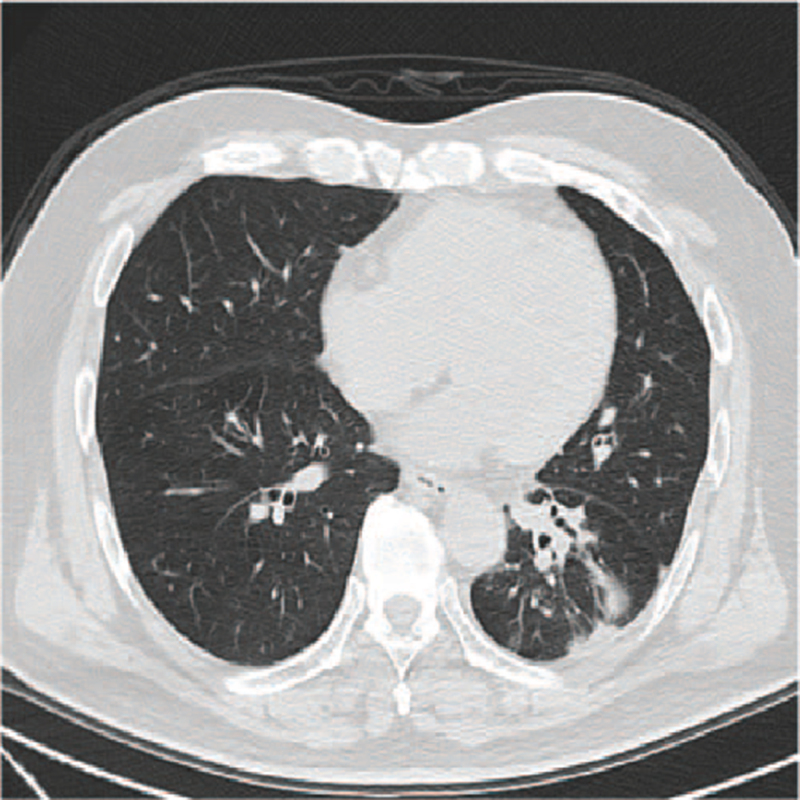
Response to chemotherapy regimen: etoposide plus cisplatin (April 2020).

**Figure 7:**
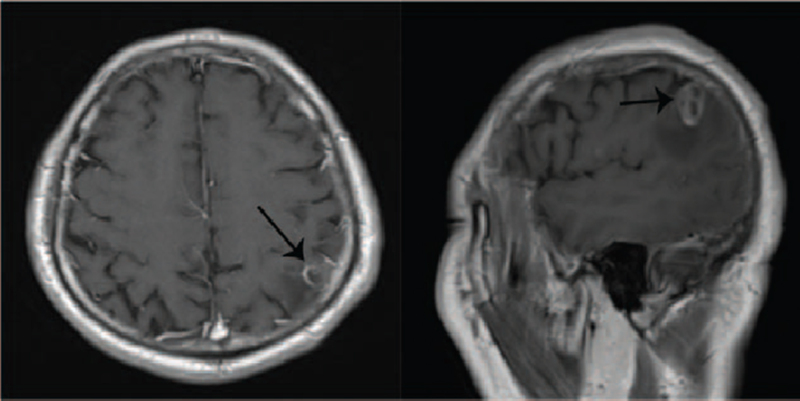
Brain MRI suggested cerebral metastases (October 2020). MRI = magnetic resonance imaging.

Icotinib (125 mg thrice daily) was administered as first-line treatment in June 2019, and the best response was partial response according to the Response Evaluation Criteria in Solid Tumors guidelines (Fig. 3). After reimaging in December 2019, CT revealed a 1.0 × 2.0 cm new enlarged supraclavicular lymph node and an enlarged left lower lobe mass (Fig. 4). Laboratory findings showed that his serum arcinoembryonic antigen level increased to 17.72 ng/mL (normal range, 0–5 ng/mL) and serum neuron-specific enolase (NSE) was raised to 48.77 ng/mL (normal range, 0–16.2 ng/mL). In January 2020, left supraclavicular lymph node biopsy was performed with ultrasound-guiding. Interestingly, the results of immunohistochemistry (IHC) staining showed positivity for TTF-1 (+), CK7 (+), napsin A (+), syn (+), and CD56 (+), with a Ki-67 (+) index of 80% of small cell carcinomas. Infiltrating adenocarcinomas and small cell carcinomas, consistent with mixed adenocarcinoma and neuroendocrine carcinoma, were also observed (Fig. 5). Next-generation sequencing revealed a T790M mutation, while the ALK gene fusion was negative, and PD-L1 was not expressed. Based on the rapid development of the supraclavicular lymph node and T790M mutation negativity, we attributed metastases to the transformed SCLC and administered etoposide (180 mg, days 1–3) plus cisplatin (45 mg, days 1–3) every 3 weeks for 1 cycle. A partial response was achieved after 4 cycles of chemotherapy, according to the Response Evaluation Criteria in Solid Tumors criteria (Fig. 6), and the serum levels of NSE were reduced to 21.21 ng/mL. As the patient could not tolerate further chemotherapy, he continued taking icotinib orally and was followed up once, 2 to 3 months later. In October 2020, brain magnetic resonance imaging suggested cerebral metastases (Fig. 7), and the patient received whole brain radiotherapy 10 times to a total dose of 30 Gy and was followed up continually. After experiencing severe lumbar vertebral pain, he was almost bedridden and was readmitted to our hospital on March 24, 2021. Magnetic resonance imaging revealed metastases of the eleventh thoracic spine and the second lumbar vertebra with a pathological fracture. The patient refused chemotherapy and only accepted supportive care, resulting in a rapid deterioration in his condition, finally leading to his death on June 3, 2021.

## 3 Discussion

SCLC transformation is 1 resistance mechanism associated with first-generation EGFR-TKIs, and is more frequent in lung adenocarcinomas with EGFR-activating mutations than in EGFR wild-type tumors.^[7–9]^ A number of mechanisms of acquired resistance to EGFR-TKI therapy in EGFR-mutant lung adenocarcinoma have been described, including the EGFR T790M mutation, EGFR amplification, MET gene amplification, PIK3CA mutation, and transformation to SCLC.^[10,11]^ However, the potential mechanism underlying SCLC phenotype conversion after TKI therapy remains unclear.

We postulated 3 mechanisms that could account for the switch between NSCLC and SCLC. First, adenocarcinoma is believed to develop from alveolar type II cells located in the alveolar surface area, whereas SCLC is derived from the neuroendocrine cells of the central airways. Several studies have shown that alveolar type II cells may be common precursors of both lung adenocarcinoma and SCLC.^[12]^ Therefore, SCLC and adenocarcinoma cells could originate from the same cancer stem cells or progenitor cells.^[13]^ Second, SCLC can result from the dedifferentiation of a previously well-defined cancer, which is similar to a mechanism known to occur in prostate cancer,^[8,14]^ while the majority of transformed SCLCs retained the original EGFR-activating mutation, supporting this mechanism. Third, it is possible that the initial tumor consisted of the combined histology of NSCLC and SCLC. As the number of NSCLC cells decreased due to treatment, the SCLC component of the initial tumor became dominant.^[9]^ Consequently, it is possible that the 2 components are both present at the initial diagnosis, but the material available for analysis was limited in our case. This phenomenon emphasizes the importance of repeat biopsies in the clinical design of treatment regimens.

A rapid increase in serum NSE may be a useful indication of transformation from adenocarcinoma to SCLC in cases that are resistant to EGFR-TKI therapy.^[15–17]^ In our case, the remarkable increase in serum levels of NSE highlighted the necessity for repeat biopsy and suggested SCLC transformation. Moreover, pro-gastrin releasing-peptide (pro-GRP) during EGFR-TKI treatment is also an indication of transformation from NSCLC to SCLC.^[18–20]^ However, little attention has been paid to the NSE and pro-GRP levels in serum, as there are no routine clinical tests for NSCLC patients. Therefore, routine and dynamic tests of NSE and pro-GRP levels in the serum may be helpful for screening patients with SCLC transformation before invasive biopsies.

In summary, we report a case of EGFR-mutant NSCLC that transformed to SCLC during treatment with EGFR-TKIs. Transformation from adenocarcinoma to SCLC may originate from a minor preexistenting SCLC cell population under the selective pressure of EGFR-TKI treatment. A secondary biopsy was important for the evaluation of genetic and histological changes and the selection of an appropriate treatment following TKI resistance. Measuring serum NSE levels in serum may be useful for early detection of SCLC transformation.

## Acknowledgments

The authors thank the patient's consent for this case report to be published.

## Author contributions

All authors made a significant contribution to the work reported, whether that is in the conception, case collection, execution, acquisition of figures, analysis and interpretation, or in all these areas; took part in drafting, revising or critically reviewing the article; gave final approval of the version to be published; have agreed on the journal to which the article has been submitted; and agree to be accountable for all aspects of the work.

**Data curation:** Yiqian Jiang, Leyi Shou, Yanhong Bao, Xiaoping Xu, Suhong An, Jianfeng Lu.

**Supervision:** Yiqian Jiang, Qingmin Guo.
